# The Deleted in Liver Cancer 1 (Dlc1) tumor suppressor is haploinsufficient for mammary gland development and epithelial cell polarity

**DOI:** 10.1186/s12885-015-1642-x

**Published:** 2015-09-09

**Authors:** Pratima Basak, Rachelle Dillon, Heather Leslie, Afshin Raouf, Michael R. A. Mowat

**Affiliations:** 1 Manitoba Institute of Cell Biology, CancerCare Manitoba, Winnipeg, MB R3E 0V9 Canada; 2 Department of Biochemistry & Medical Genetics, University of Manitoba, Winnipeg, MB Canada; 3 Department of Immunology, University of Manitoba, Winnipeg, MB Canada; 4 Regenerative Medicine Program, University of Manitoba, Winnipeg, MB Canada

## Abstract

**Background:**

Deleted in Liver Cancer 1 (Dlc1) is a tumor suppressor gene, which maps to human chromosome 8p21-22 and is found frequently deleted in many cancers including breast cancer. The promoter of the remaining allele is often found methylated. The Dlc1 gene encodes a RhoGAP protein that regulates cell proliferation, migration and inhibits cell growth and invasion when restored in Dlc1 deficient tumor cell lines. This study focuses on determining the role of Dlc1 in normal mammary gland development and epithelial cell polarity in a Dlc1 gene trapped (gt) mouse.

**Methods:**

Mammary gland whole mount preparations from 10-week virgin heterozygous Dlc1^gt/+^ gene-trapped mice were compared with age-matched wild type (WT) controls. Hematoxylin-Eosin (H&E) and Masson’s Trichrome staining of histological sections were carried out. Mammary glands from Dlc1^gt/+^ mice and WT controls were enzymatically digested with collagenase and dispase and then cultured overnight to deplete hematopoietic and endothelial cells. The single cell suspensions were then cultured in Matrigel for 12 days. To knockdown Dlc1 expression, primary WT mammary epithelial cells were infected with short hairpin (sh) RNA expressing lentivirus or with a scrambled shRNA control.

**Results:**

Dlc1^gt/+^ mice showed anomalies in the mammary gland that included increased ductal branching and deformities in terminal end buds and branch points. Compared to the WT controls, Masson’s Trichrome staining showed a thickened stromal layer with increased collagen deposition in mammary glands from Dlc1^gt/+^ mice. Dlc1^gt/+^ primary mammary epithelial cells formed increased solid acinar spheres in contrast with WT and scrambled shRNA control cells, which mostly formed hollow acinar structures when plated in 3D Matrigel cultures. These solid acinar structures were similar to the acinar structures formed when Dlc1 gene expression was knocked down in WT mammary cells by shRNA lentiviral transduction. The solid acinar structures were not due to a defect in apoptosis as determined by a lack of detectible cleaved caspase 3 antibody staining. Primary mammary cells from Dlc1^gt/+^ mice showed increased RhoA activity compared with WT cells.

**Conclusions:**

The results illustrate that decreased Dlc1 expression can disrupt the normal cell polarization and mammary ductal branching. Altogether this study suggests that Dlc1 plays a role in maintaining normal mammary epithelial cell polarity and that Dlc1 is haploinsufficient.

## Background

Breast tumors undergo frequent gene copy number changes [1, 2]. One chromosomal region, 8p22, shows frequent copy number loss in 16–20 % of breast cancers, without a loss of heterozygosity, suggesting the location of a haploinsufficient tumor suppressor gene(s) (ibid.). The Deleted in Liver Cancer-1 (Dlc1) tumor suppressor gene maps to this chromosomal region (for review see [3]). The Dlc1 gene was initially found associated with frequent deletions in hepatocellular carcinomas [4]. Using tiling microarrays, Xue et al. showed that heterozygous deletion of Dlc1 occurred in approximately 50 % of breast, liver, pancreatic and lung tumors and more than 70 % of colon cancers [5]. Although these deletions could be up to five Mbps (~20 genes), they always included the Dlc1 locus (ibid.). The promoter of the remaining allele of Dlc1 is also frequently found hypermethylated in many cancer types [6]. Chromosome region 8p22 contains several tumor suppressor genes that may cooperate with Dlc1 loss to increase tumor aggressiveness [7]. Reduced or absent expression of Dlc1 has been frequently found in primary breast tumors and cell lines [8, 9]. Transfection of Dlc1 into deficient breast tumor cells will inhibit both *in vitro* and *in vivo* tumor cell growth [9, 10]. Another study, using matched malignant and nonmalignant human breast cancer cell lines, showed that the nonmalignant line had Dlc1 transcript levels 3-fold greater than the malignant clone [11]. Overall these results suggest that Dlc1 may be an important tumor suppressor in breast cancer.

The Dlc1 protein shows homology with the rat p122^RhoGAP^ protein, which was initially found as a binding partner of Phospholipase C-delta 1 (PLC-δ1), stimulating its activity [12]. The Dlc1 RhoGAP protein has three structural regions namely; an amino terminal SAM2 (sterile α motif), a Rho GTPase activating protein (RhoGAP) and a StAR related lipid transfer (START) domains [3]. Dlc1 protein shows strong Gap activity for RhoA, B and C [12–14]. The Dlc1 protein has been localized to caveolae and binds to caveolin 1 [15–17]. Also, the Dlc1 protein has been found in focal adhesions binding to adhesion proteins vinculin [18] and tensin [19–21]. Dlc1 has also been found to bind FAK (focal adhesion kinase) and talin with this binding region being needed for its full tumor suppressor activity *in vitro* [22]. This region when mutated does not interfere with Dlc1’s RhoGAP activity, indicating that signalling pathways other than Rho may also be needed for its tumor suppressor activity (ibid.).

Postnatally the mouse mammary gland develops through branching morphogenesis to form a treelike ductal system that penetrates into the stromal fat pad followed by alveologenesis during pregnancy (for review see [23]). The key structure driving this process is the terminal end bud (TEB) where epithelial precursors grow and differentiate into luminal and myoepithelial cell compartments forming the bilayered duct [24]. As in all epithelial tissues, the establishment of polarity by epithelial cells is critical for proper lumen and ductal formation influenced by interactions with the extracellular matrix and cell-cell adhesions [25]. Mammary epithelial cell polarity can be modelled *in vitro* by culturing cells in a laminin-rich extracellular matrix (Matrigel), which allows formation of hollow spherical acinar structures [26]. The loss of this cell polarity is a characteristic feature of advanced epithelial tumors and may play an important role in their initiation and progression [27].

The Rho GTPases play critical roles in the formation and maintenance of epithelial cell adhesion structures [28]. Several studies have shown the importance of Rho signalling in mammary gland development. Heterozygous loss of the p190B RhoGAP gene results in delayed ductal growth, due to reduced terminal cap cell layer proliferation compared with wild type mice [29]. Transplantation of homozygous p190B^-/-^ mammary anlagen resulted in no ductal outgrowth (ibid.). The closely related p190A RhoGAP also shows halpoinsufficiency, with a slight delay of ductal outgrowth and a disrupted TEB architecture [30]. Tissue transplants showed that p190A was needed in both the epithelial and stromal cell layers for ductal outgrowth, although the phenotype was less severe than p190B deficiency (ibid.). Constitutive expression of Vav2, a guanine nucleotide exchange factor for Cdc42 and Rac1, results in disruption of the acinar architecture in mammary cell line 3D cultures [31]. In contrast, RhoA activation was associated with stability of acinar structures and E-cadherin cell-cell adhesions (ibid.).

To understand the role that Dlc1 loss plays in breast cancer, it is important to understand its role in normal mammary morphogenesis. Also, since Rho signalling is important for mammary gland morphogenesis, we wanted to learn if Dlc1 played a role in mouse mammary ductal development. To carry out these experiments, we made use of a gene trapped (gt) Dlc1 mouse that was hypomorphic for Dlc1 isoform 2 expression [32]. This mouse shows embryonic lethality when homozygous, but appears “normal” when heterozygous (ibid.). In the present study, we showed that these heterozygous Dlc1^gt/+^ mice exhibited anomalies in mammary gland with increased ductal branching and irregularity in the branch points/terminal end buds. The normal polarization and lumen formation that occurs during epithelial cell morphogenesis in 3D acinar cultures were also affected as a result of heterozygous loss of Dlc1. Knockdown of Dlc1 expression in wild type cells showed a similar loss of polarity. These results suggest that Dlc1 plays a role in maintaining normal mammary epithelial cell polarity and also is haploinsufficient for mammary ductal development.

## Methods

### Animals

The generation of the Dlc1 gene trapped mutant mice (Dlc1^Gt(XE082)Byg/+^) were previously described [32]. The Dlc-1^gt/+^ mice were backcrossed to C57Bl/6 mice for at least 7 generations before experiments were carried out.

### Animal ethics

All experiments were performed as per the Canadian Council on Animal Care (CCAC) and were affirmed by the University of Manitoba Animal Protocol Management and Review Committee before experimentation.

### Whole mount preparation of mammary gland

For the whole gland morphological analysis, the fourth inguinal mammary glands were surgically removed from 10 week old heterozygous Dlc1^gt/+^ gene trapped and wild type virgin female mice. The whole mounts of mammary glands were prepared as previously described [33]. Briefly, mammary glands were fixed in 4 % paraformaldehyde, defatted in acetone, dehydrated in ethanol, followed by staining with 0.2 % carmine alum overnight. The whole mounts were then destained, ethanol dehydrated and finally cleared in xylene. The whole mounts were analyzed by light microscopy for various parameters including branching morphogenesis, number of TEBs or branch points and branching density. Individual whole mount glands were divided into two regions, proximal and distal relative to the lymph node, for counting the TEBs for each region and summed to obtain the total number of TEBs/individual mammary gland. Abnormal or defective TEBs were defined as TEBs that were trifurcated or had multiple buds on the neck. The thickening of the ductal branches in the mammary glands was determined using the measuring tool in Adobe Acrobat X Pro.

### Immunofluorescent staining of mouse mammary glands

Paraffin embedded histological sections of mouse mammary glands were used for immunofluorescence staining. The slides were deparaffinised and hydrated by washing with xylene (twice), 100 % EtOH, 90 % EtOH, 70 % EtOH and water for 5 mins. Antigen retrieval was carried out by boiling the slides for 15–20 min in citrate buffer and allowed to cool at room temperature for 15 min. After incubation in water the slides were permeabilized with 0.5 % Triton X-100. After two PBS washes, sections were blocked with 10 % goat serum for 2 h. Then primary antibody was added to the slides and incubated overnight at 4 °C in a humidified chamber. Primary antibodies used were cytokeratin 18 (Abcam, at 1:300 dilution), and cytokeratin 14 (Covance, CA; 1:400), cleaved caspase -3 (Cell Signaling Technology, 1:200), Ki67 (Abcam, 1:200). The expression of each protein was detected using either FITC, PE or Cy3 conjugated secondary antibodies (at 1:300 dilution). DAPI (Sigma, USA) or Topro-3 (5 μM, Molecular Probes, Eugene, OR) were used to stain the nculeus.

### Dissociation and preparation of mammary single cell suspensions

For the preparation of mammary single cell suspensions, fourth inguinal mammary glands from 10 week old virgin WT or Dlc1^gt/+^ mice were surgically removed and mechanically chopped followed by enzymatic dissociation with collagenase (US Biologicals, Swampscott, MA) and dispase (Life Technologies, Burlington ON) for 2–3 h at 37 °C as described [34]. Briefly, the digested suspensions were pelleted, resuspended in 1 mM EDTA-PBS buffer and the number of viable cells determined by automated cell counter (Bio-Rad TC10) according to the manufacturer’s instructions. Dissociated mammary epithelial cells were cultured overnight with mammary epithelial growth media, (MEGM; Dulbecco’s Modified Eagle media (Gibco), 5 μg/mL insulin (Sigma), 1 μg/mL hydrocortisone (Sigma), 10 ng/mL epidermal growth factor (EGF; BD Biosciences), 1× Penicillin/Streptomycin (P/S; Life Technologies), 35 μg/ml bovine pituitary extract (BD Bioscience, San Jose, CA)] supplemented with 5 % FBS (BD Biosciences, San Jose, CA) in 6-well plates to allow depletion of hematological and endothelial cells.

### 3D Matrigel culture of the mammary epithelial cells

Adherent mammary single cells obtained after overnight culture were then trypsinized and counted. Approximately 2 × 10^5^ viable cells were placed in 8-well chamber slides containing growth factor reduced Matrigel (Corning, VWR Edmonton, AB) with cell culture media consisting of MEGM supplemented with 2 % FBS as described [33, 34]. The media was changed every 2–3 days and maintained for 10–12 days at which point the acinar structures were fixed, permeabilized and stained as described below.

### Histological analyses

For histological analyses, the fourth inguinal mammary glands from 10 week old virgin WT or Dlc1^gt/+^ mice were surgically removed and fixed overnight at 4 °C with phosphate-buffered 4 % paraformaldehyde. Paraffin embedding, sectioning, H&E and Trichrome staining were performed by the Manitoba Tumor Bank Histology Core Facility (CancerCare Manitoba). Thereafter, H&E and Trichrome stained sections were analysed and imaged on the EVOS XL cell imaging system (Life Technologies) according to the manufacturer’s instructions.

### Short Hairpin RNA (shRNA) lentiviral transduction

Primary mammary epithelial cells from 10 week old WT virgin mice were infected with a pool of lentivirus produced from two pGIPZ-puro shRNA expression vectors targeting Dlc1 (Thermo Fisher, St Louis, MO), as previously described [35]. For the knockdown experiments, a pGIPZ-puro shRNA scrambled control was also used. The infected cells were selected with 1.5 μg/ml puromycin 2 days after infection. To obtain cells containing stably integrated shRNA, the puromycin selection was continued for at least 2 weeks. Lentiviral packaging plasmids pCMV-dR8.2 and pCMV-VSVG (Addgene plasmids #8455 for pCMV-dR8.2 and #8454 for pCMV-VSVG) were used to co-transfect with each plasmid into HEK 293T cells for virus production [36]. The viral supernatant was concentrated by ultracentrifugation and lentivirus transduction was performed using a multiplicity of infection (MOI) of approximately 10.

### RNA isolation and RT-qPCR analysis

RNA was extracted from puromycin selected lentivirus infected mammary epithelial cells using Trizol (Life Technologies, Burlington, ON) according to the manufacturer’s protocol. Biorad CFX real-time PCR system was used to determine the relative mRNA expression levels using the ΔCT method and all values were normalized to GAPDH expression. Sequence of primers used; GAPDH forward 5′-GCACAGTCAAGGCCGAGAAT-3′, reverse 5′-GCCTTCTCCATGGTGGTGAA-3′; Dlc1 forward 5′-CGGTTGTTGCTAGAGCCTTG-3′, reverse 5′- ACCTAAGACAGACAGGAAGCAG-3′.

### Western blot analysis

Total proteins were extracted from primary mammary epithelial cells of Dlc1^gt/+^ and WT mice and 70 μg of lysates were separated by SDS-PAGE. The proteins were then transferred to Immobilon-P PVDF (polyvinylidene difluoride) membrane (Millipore, ON] for determination of specific protein expression levels using anti-rabbit Dlc1 (Santa Cruz Biotechnology, Dallas, TX,1:1000, cat#sc-32931,] using chemiluminescence as described by [32]. The blots were visualized by incubation with SuperSignal West Femto Substrate (Thermo Scientific, Rockford, IL) in the Fusion FX Gel Documentation system (Vilber Lourmat, Germany). The signal intensities were determined using the Fusion-CAPT software (Vilber Lourmat, Montreal Biotech, Dorval, PQ), and Dlc1 protein expression levels were determined as a ratio to β-actin (1:10,000 dilution, Sigma, St. Louis, MO).

### Confocal microscopy

The media was aspirated from each well of the 8-well chamber slide and the acinar structures fixed with 4 % paraformaldehyde at room temperature for 20 min. In some experiments, acinar structures were treated with 250 μM etoposide (Sigma) for 24 h and then fixed. Once fixed, the wells were rinsed with PBS and permeabilization was carried out using 0.5 % Triton X-100 for 10 min at 4 °C. Then they were rinsed thrice with PBS containing 100 mM glycine. The acinar structures were blocked with IF buffer [34, 37] (130 mM NaCl, 7 mM Na_2_HPO_4_, 3.5 mM NaH_2_PO_4_, 0.1 % BSA, 0.2 % Triton X-100 and 0.05 % Tween-20) containing 10 % goat serum [37] for 60 min. Structures were then incubated with primary antibodies specific for β-catenin (BD Biosciences, San Jose, USA Cat# 610153), α-6 integrin (Millipore, Cat# MAB1378) and E-cadherin (BD Biosciences, San Jose, USA, Cat# 610181) cleaved caspase-3 (Cell signaling technology, Beverly, MA,) overnight at 4 °C. The acinar structures were then carefully rinsed 2–3 times for 20 min with IF buffer at room temperature with gentle rocking followed by incubation with fluorescent-tagged secondary antibodies conjugated to FITC or PE (BD Biosciences) (1:300) for 1 h at room temperature. After rinsing the structures with one wash of IF buffer and 2–3 washes of PBS, the nuclei were counterstained with To-Pro-3 (5 μM, Molecular Probes, Eugene, OR)or DAPI (4′,6-diamidino-2-phenylindole) (Sigma). After a final rinse in PBS for 5 min at room temperature, the chamber slide was mounted with a glass cover slip using Prolong Anti-fade reagent (Life Technologies, Burlington, ON) and allowed to dry overnight at room temperature. Microscopic analysis was performed using a FV500 laser scanning confocal microscopy system (Olympus) and Z stacking function was used for serial confocal sectioning of the acinar structures at 2 μm intervals. Images were acquired using Fluoview software [38].

### Rho activity assay

Primary mammary epithelial cells from WT and Dlc1^gt/+^ mice were grown to confluency on collagen coated 10 cm cell culture plates. The cells were then serum starved for 1 h followed by stimulation with 15 % serum for 5 min. Soon after this stimulation, the cells were washed twice with ice-cold Tris Buffered Saline (TBS). The entire procedure was performed in the cold room. Cold lysis buffer composition, as described by Ren and Schwartz [39], was added to the cells. The cell lysates were prepared and analysed for active RhoA by the pull down assay as described by Ren and Schwartz [39]. Briefly, cells were scraped rapidly to avoid nuclear lysis and the lysates were transferred to 1.5 ml tubes and spun at 13,000 g for 10 min at 4 °C. Cleared lysates were transferred to tubes containing 30 μg of GST-Rhotekin-Rho binding domain glutathione-Sepharose beads and rotated at 4 °C for 1 h. The beads were then washed four times at 5000 rpm (Sorvall Legend Micro21R centrifuge, Thermoscientific) for 15 s with 600 μl cold Tris buffer. The beads were then resuspended in SDS sample buffer containing 40 mM dithiothreitol. Following SDS-PAGE and Western blotting, the membranes were probed with anti-RhoA antibody (Cell Signaling Technology, Beverly, MA, Cat#2117,). The signal intensities were determined using the Fusion-CAPT software (Vilber Lourmat) and active Rho signal intensity was normalized to total Rho.

### Statistical analysis

The two tailed student *T*-test was used to determine statistical significance (*p* < 0.05) using the GraphPad Prism 4.02 program (San Diego, CA).

## Results

### Heterozygous Dlc1 loss affects mammary gland branching

**Fig. 1:**
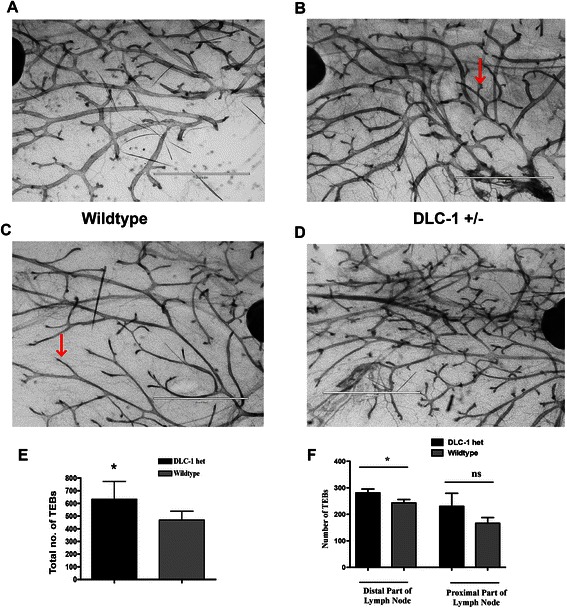
Increased mammary terminal end buds/branch points in Dlc1^gt/+^ mice. **a**–**d** Representative images of fourth inguinal mammary gland whole mounts of 10 week old virgin wild type mice (**a** & **c**) and Dlc1^gt/+^ gene-trapped mice (**b** & **d**) on C57BL/6 background. Red arrowhead indicates terminal end buds (TEBs). Scale bar, 2 mm. **e** Bar graph showing that the total number of TEBs in 10 week old Dlc1^gt/+^ compared to the wild type mammary glands. Data represents the mean ± SD of 5 different glands (*N* = 5) from 5 different mice for each group. **f** Quantification of the number of TEBs in the distal and proximal regions relative to the central lymph node. Data represents the mean ± SD. A total number of 5 different glands from 5 different mice were studied for each group by two-tailed Student’s *t* test, *p < 0.05. ns-not significant

Previous results had suggested that the chromosome region 8p22, where Dlc1 maps, may contain a haploinsufficient tumor suppressor gene in breast cancer [1]. Therefore, we wanted to determine whether heterozygous loss of Dlc1 in a mouse model would affect mammary gland development. To do so, we made use of a Dlc1 gene trapped mouse model that we had previously developed [32]. Although the homozygous Dlc1 gene trapped mice show embryonic lethality, the heterozygous mice are grossly normal. To evaluate the effect of heterozygous loss of Dlc1 on mammary gland development, we examined whole mount mammary gland preparations from these mice. On comparing the mammary glands from the age matched WT and heterozygous Dlc1^gt/+^ gene-trapped mice, we found that the WT mammary gland showed regular ductal branching as is observed in virgin females of the C57BL background [Fig. 1a–d]. As has been previously reported [40], we also observed limited alveolar structures or secondary branching from the main ductal branches in regions proximal and distal to the central lymph node in WT mammary glands [Fig. 1a and c]. Unlike WT control mammary glands, Dlc1^gt/+^ mammary glands showed increased ductal branching and side branching from the main ducts [Fig. 1b, d and e]. Furthermore, mammary glands from Dlc1^gt/+^ mice also showed a significant increase in the total number of TEBs or branch points in the region distal to the central lymph node [Fig. 1f].

### Deformity in the terminal end buds or branch points due to Dlc1 deletion

**Fig. 2:**
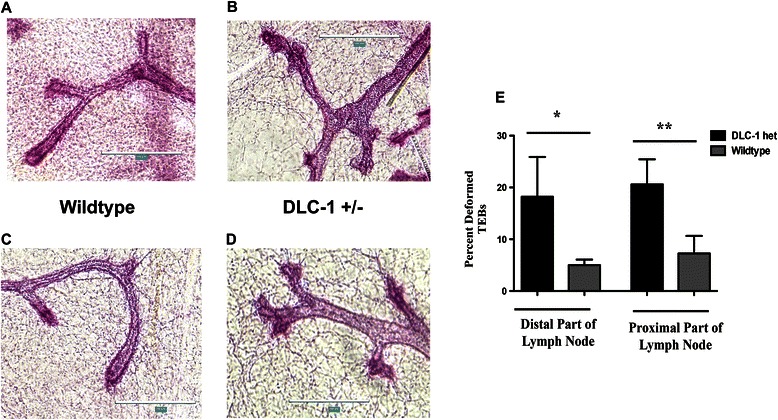
Terminal end bud deformities in Dlc1^gt\+^ mammary glands. **a** & **c** Representative images of the fourth inguinal mammary gland whole mounts from 10-week-old virgin female wild type (**a** & **c**) and Dlc1^gt/+^ (**b** & **d**) mice stained with carmine alum. Scale bar 200 μm. **e** Bar graph quantifying the number of deformed TEBs comparing distal or proximal regions relative to the central lymph node. Data represent the mean ± SD of 5 different glands (*N* = 5) from 5 different mice studied for each group. **p < 0.01, *p < 0.05

When the whole mounts were examined closely, we found branch points/TEB structures with smooth bulbous structures in WT mice [Fig. 2a and c]. In contrast, the Dlc1^gt/+^ mice displayed more ductal side branching and deformed or irregular bulbous structures at the branch points [Fig. 2b and d]. We noted a significant increase in the number of deformed TEBs and branch points in both the proximal and distal parts of the glands from the Dlc1^gt/+^ mice when compared with the age-matched WT mice [Fig. 2e].

**Fig. 3:**
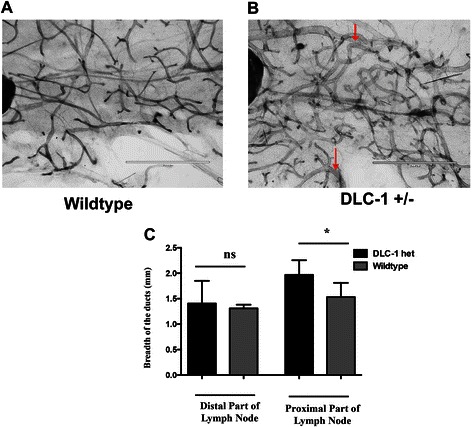
Mammary ductal thickening in Dlc^gt/+^ mice. **a** & **b** Representative images of the fourth inguinal mammary gland whole mounts from 10-week-old virgin female wild type mice (**a**) and heterozygous Dlc1^gt/+^ gene trapped mice (**b**). Red arrows indicate ductal thickening. Scale bar, 2 mm. **c** Quantification of ductal thickness. The bar graph shows that the average width of ductul branches from the proximal region compared with the distal region relative to the central lymph node. Data represent the mean ± SD from a total number of 5 different glands (*N* = 5) from 5 different mice for each group. **p < 0.01, *p < 0.05

The mammary glands from Dlc1^gt/+^ mice displayed increased thickening of the ductal branches compared with WT controls [Fig. 3a and b]. The significant increase in ductal branch thickening (red arrowheads) in Dlc1^gt/+^ mice was observed specifically in the proximal part of the mammary gland, but not in the distal region [Fig. 3c].

We have not observed any evidence of hyperplasia or spontaneous tumors in the Dlc1^gt/+^ mice kept for one year or more.

### Heterozygous Dlc1^gt/+^ gene-trapped mice showed increased stromal layer thickening surrounding alveolar and duct structures

**Fig. 4:**
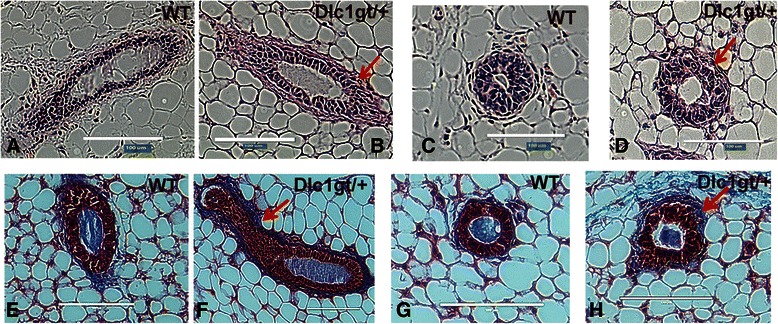
Dlc1^gt/+^ mice show increased stromal collagen deposition in mammary glands. **a**–**d** Histological sections stained with H&E showed a thickened stromal layer in both the alveolar and ductal structures in the mammary glands from 10-week-old Dlc1^gt/+^ (**b** & **d**) compared with WT mice (**a** & **c**). These figures are representative of a total of 4 different glands (*N* = 4) from 4 different mice studied for each group. **e**–**h** Representative Masson’s trichrome staining (*blue*) of mammary glands from 10 week old virgin WT (**e** & **g**) and Dlc1^gt/+^ mice (**f** & **h**). The red arrows indicate areas of collagen deposition into the surrounding stroma. The fourth inguinal mammary glands from 3 different mice from each genotype were studied. Scale bar 100 μm

Hematoxylin and Eosin stained sections showed the presence of a thickened stromal layer surrounding both the alveoli and ducts in Dlc1^gt/+^ mice compared with age-matched controls [Fig. 4a–d]. In order to determine whether this was due to stromal collagen deposition, we stained sections with Masson’s Trichrome [Fig. 4e–h]. It was observed that the Dlc1^gt/+^ mammary gland sections of both ducts [Fig. 4f] and alveoli [Fig. 4h] had increased collagen-rich staining (blue colour) in the extracellular matrix.

**Fig. 5:**
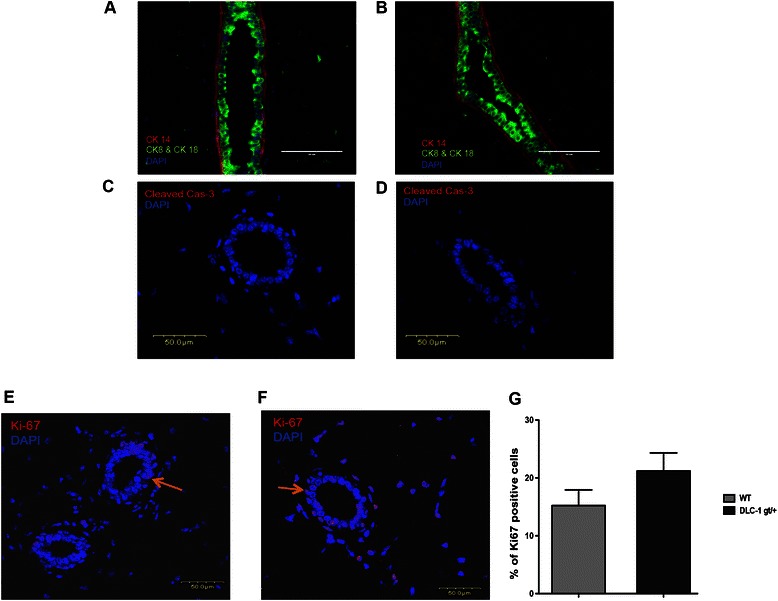
Histological sections of mouse mammary glands stained with cytokeratin lineage markers, cleaved caspase-3 and Ki67. **a**–**b** Immunostaining of the mouse mammary glands with lineage markers cytokeratins 14 (Basal/myoepithelial) and 18 (Luminal) from 10-week old WT mice (**a**) and Dlc1^gt/+^ mice (**b**). Cleaved caspase-3 staining in 10-week old Dlc1^gt/+^ (**d**) and WT mice (**c**). Ki67 staining of mouse mammary glands from WT (**e**) and Dlc1^gt/+^ (**f**). The orange arrows indicate cells with Ki67 positive nuclei. The bar graph is the percentage of Ki67 positive cells (**g**). Data represent the mean ± SEM from a total number of 3 different glands (*N* = 3) from 3 different mice for each group *p* = 0.2. Scale bar 50 μm

Histological sections of mouse mammary glands were stained with lineage markers cytokeratins 14 (CK-14) and 18 (CK18). We found no visible differences in the Dlc1^gt/+^ mice mammary glands compared to the age matched control mice [Fig. 5a and b]. The ducts showed normal myoepithelial or basal (CK14) and luminal (CK18) epithelial cell organization. We also could not detect any cleaved caspase-3 staining in the two genotypes [Fig. 5c and d]. We also quantified the Ki67 positive cells (orange arrow) in the histological sections and found no statistically significant difference in cells in S-phase between the two genotypes [Fig. 5e–g].

### Loss of Dlc1 affects epithelial cell polarity in mammary epithelial cells cultured in 3D Matrigel

**Fig. 6:**
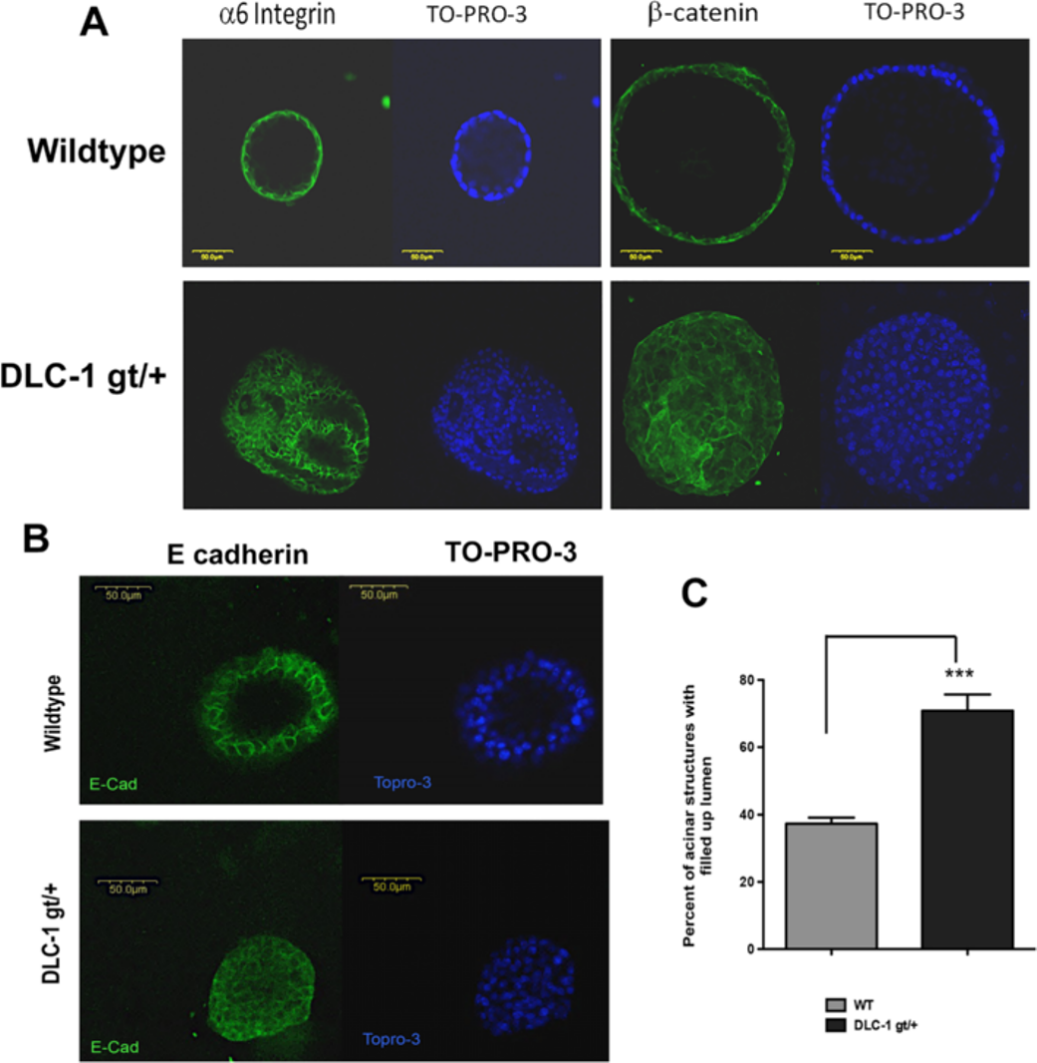
Mammary epithelial cells from Dlc1^gt/+^ mice show defects in acinar lumen formation when grown in Matrigel cultures. **a** Representative images of acinar structures formed from mammary epithelial cells from 10 week old wild type (*top*) and Dlc1^gt/+^ (*lower*) mice grown in 3D Matrigel cultures. Acini are shown at day 12 after plating. Acinar structures were stained with To-Pro-3 and antibodies against α6 integrin (*left*), or β-catenin (*right*). **b** Acinar structures were stained with E-Cadherin and To-Pro-3 from 10 week old wild type (*top*) and Dlc1^gt/+^ (*lower*). Scale bar, 50 μm. Serial confocal images were taken using Z-stacking function through the middle of the acini. **c** Quantification of acini showing filled lumens. Data is the mean ± SD from a total number of 5 different glands (*N* = 5) from 5 different mice for each. **p < 0.001

One potential explanation for the changes in ductal branching observed in Dlc1^gt/-^ mice is that the polarized architecture of the epithelial cells is compromised. In order to examine this possibility we isolated mammary epithelial cells from Dlc1^gt/+^ and age-matched WT control mice and cultured them in Matrigel. The cells were allowed to grow for 10–12 days and then fixed and stained. The acinar structures were stained with polarity markers α6 integrin (basolateral marker), β-catenin (cell adhesions) and E-cadherin (adherent junctions). The mammary epithelial cells from wild type C57BL mice showed acinar-like structures with hollow lumen in the 3D Matrigel cultures [Fig. 6a, upper] with proper distribution of α6 integrin, β-catenin [Fig. 6a, upper] and E-cadherin [Fig. 6b upper]. In contrast, we found that Dlc1^gt/+^ mammary epithelial cells showed defective acinar morphogenesis with over 70 % exhibiting filled acinar structures [Fig. 6a, lower; b, lower; c].

**Fig. 7:**
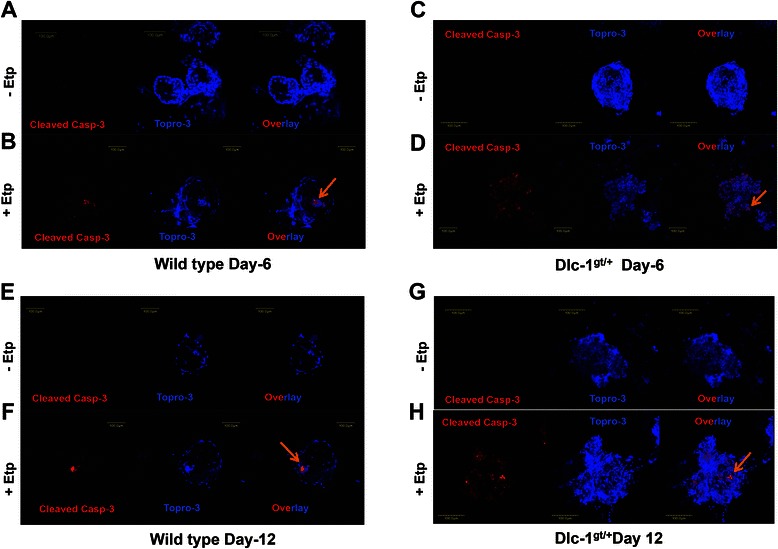
Luminal filling of the acinar structures formed from mammary epithelial cells of Dlc1^gt/+^ mice grown in 3D Matrigel culture is not due to defects in apoptosis. Representative images of acinar structures from mammary epithelial cells from 10 week old wild type mice (**a** & **e**) at days 6 & 12 after culturing in Matrigel or treated with etoposide (**b** & **f**) and stained with cleaved caspase-3 antibody and To-Pro-3. Acinar structures of mammary epithelial cells from Dlc1^gt/+^ mice at day 6 (**c**) and day 12 (**g**) or treated with etoposide (**d** & **h**). Scale bar 100 μm

One possible reason for the filled acinar structures in cells from Dlc1^gt/+^ mice is a defect in apoptosis. To test this possibility, we stained mammary acinar structures with an antibody for cleaved capapse-3. Acinar structures at day 6 did not show positivity for cleaved caspase-3 [Fig. 7a]. Whereas treatment with etoposide, to induce apoptosis, showed evidence of caspase-3 cleavage [Fig. 7b]. Similarly, the WT cells cultured for 12 days showed no positivity for cleaved caspase-3, but did after etoposide treatment [Fig. 7e, f]. The acinar structures from Dlc1^gt/+^ mammary epithelial cells showed filled acinar structures at day 6 [Fig. 7c] and at day 12 [Fig. 7g]. The filled acinar structures from Dlc1^gt/+^ mice did not show any cleaved caspase-3 positivity but did show significant amounts following etoposide treatment [Fig. 7d and h].

**Fig. 8:**
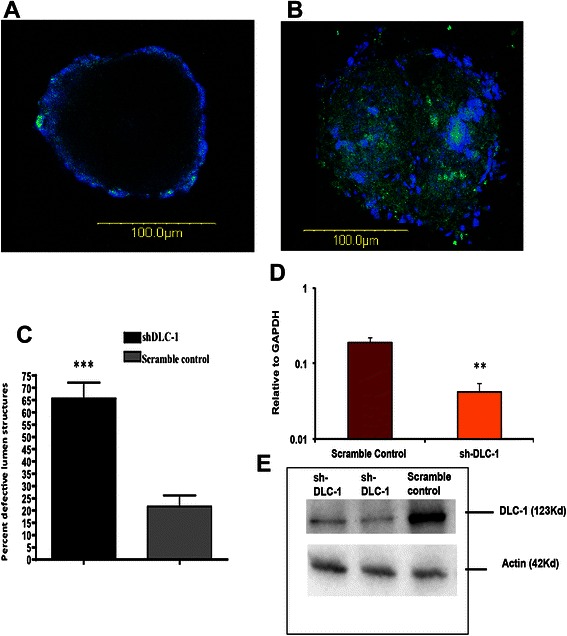
Knockdown of Dlc1 in primary mammary epithelial cells show defects in acinar lumen formation. **a**–**b** Merged images showing green fluorescence protein expressed from the shRNA lentiviral vectors and DAPI (*blue*) in 3D Matrigel cultures. WT mammary epithelial cells infected with lentivirus expressing scramble control (**a**) or Dlc1 shRNA (**b**). Scale bar 100 μm. **c** Bar graph showing the percentage of solid acinar structures after lentivirus infection (*p* < 0.0001). **d** Quantitative RT-PCR analysis of Dlc1 mRNA levels following infection with Dlc1 targeting and scrambled control lentiviruses. Transcript levels were normalized to GAPDH (*p* = 0.0024). **e** Immunoblot analysis of Dlc1 protein levels in WT mammary epithelial cells infected with Dlc1 targeting and scrambled control lentiviruses. Four independent experiments with *n* = 3 animals in each group were used in this study

To confirm the finding of defective acini in epithelial cells from Dlc1^gt/+^ mice, we knocked down Dlc1 expression using shRNA in primary mouse wild type mammary epithelial cells using lentivirus[Fig. 8]. Knockdown of Dlc1 in primary mammary epithelial cells was confirmed by real time RT-PCR and Western blotting [Fig. 8d and e]. Furthermore, we found that similar to mammary epithelial cells from Dlc1^gt/+^ mice, the knockdown of endogenous Dlc1 expression led to a significant increase in irregular acinar-like structures with filled lumen compared with scrambled shRNA control [Fig. 8c].

### Primary mammary epithelial cells from heterozygous Dlc1^gt/+^ gene-trapped mice cells show increased RhoA activity

**Fig. 9:**
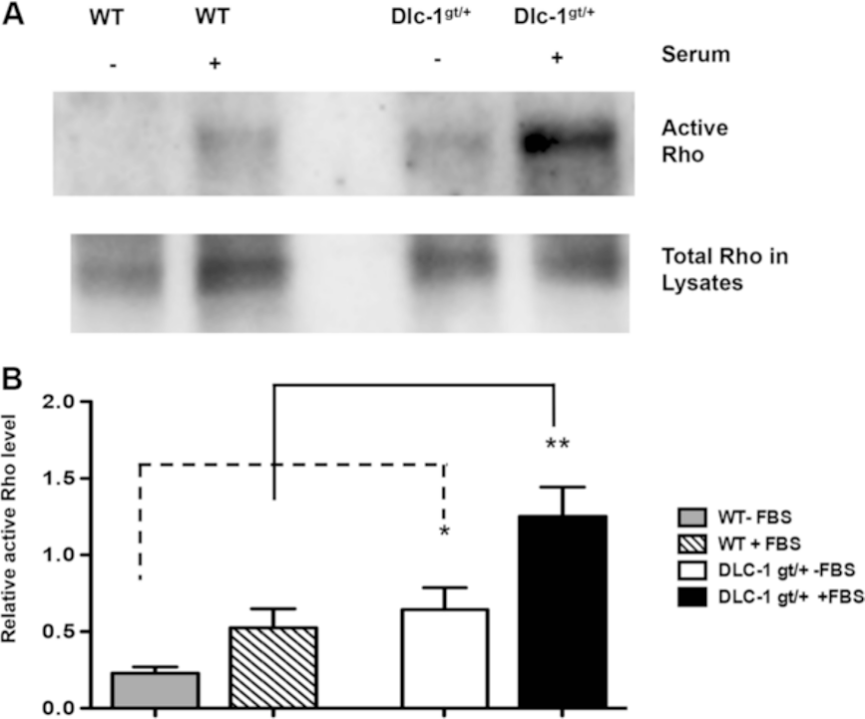
Rho Activity assay in primary mouse mammary epithelial cells. **a** Representative western blot showing the activation of RhoA in primary mammary epithelial cells plus or minus serum addition as described in the materials and methods section. **b** Bar graph showing the active RhoA levels determined by normalizing the amount of RBD-bound Rho to the total RhoA in whole cell lysates. Data is represented as the mean ± SEM from a total of 6 independent experiments. Statistical analyses were performed using student *T* test. *p < 0.05; **p < 0.01. [WT - FBS vs WT+FBS, *p* = 0.04; Dlc1^gt/+^-FBS vs Dlc1^gt/+^+FBS, *p* = 0.022; WT-FBS vs Dlc1^gt/+^ -FBS, *p* = 0.021; WT+FBS vs Dlc1^gt/+^+FBS, *p* = 0.0085]

Next, we wanted to determine whether the loss of one Dlc1 allele resulted in increased RhoA activity. A Rho-GTP pull down assay was performed to evaluate the active Rho levels in primary mammary epithelial cells. We found that cells from Dlc1^gt/+^ mice showed significant elevation in RhoA activity both before and after stimulation with serum compared with wild type mammary epithelial cells [Fig. 9].

## Discussion

It has been previously shown that Rho signalling plays an important role in controlling mammary ductal morphogenesis [29–31]. Our results showed that heterozygous loss of Dlc1 increases ductal branching. This is in contrast to what was seen in mice with heterozygous or homozygous loss of p190B RhoGAP, which resulted in decreased numbers and rate of ductal outgrowth [29]. This suggests that it is the local temporal control of Rho activity and not just the increase in Rho activity that is critical for disrupting ductal morphogenesis. For example, p190B RhoGAP plays an important role in cytokinesis by controlling Rho activity at the cleavage furrow [41] whereas, Dlc1 controls focal adhesion dynamics and cell motility [32, 42–44]. Another study using Net1 RhoGEF knockout mice showed delayed ductal extension and reduced ductal branching in the mammary gland, which supports the contention that precise control of RhoA activation is essential for proper mammary morphogenesis [45].

Mouse strain differences in mammary ductal side branching pattern is dictated by the stromal compartment [46]. Although the heterozygous loss of Dlc1 would be found in both the stromal and epithelial layers, we have shown that this effect is at least partially due to defects in epithelial cell polarity, as revealed in 3D cultures. In this study, we also found increased stromal collagen deposition surrounding the ducts and alveolar structures. Previously, it was shown that targeted activation of the Rho effector ROCK2 kinase to mouse skin resulted in increased collagen deposition [47]. Whether the increased collagen deposition is due to altered Rho activity in stromal or epithelial cells will be the subject of future experiments.

One of the most intriguing findings of our work is the filling up of the lumen when mammary epithelial cells from the Dlc1^gt/+^ mice were placed in 3D Matrigel cultures. This phenotype was also seen when Dlc1 was knocked down in wild type mammary epithelial cells. Our results indicate that this phenotype is not due to a defect in apoptosis, as no cleaved caspase 3 was detected during lumen formation in wild type or Dlc1^gt/+^ 3D cultures. This is in agreement with other studies in which no induction of apoptosis was seen during lumen formation in primary mouse mammary epithelial cells [48]. Although lumen filling is an extreme phenotype, the mammary ducts did not show evidence of hyperplasia of epithelial cells lining the ducts or acini. This may indicate that when Dlc1^gt/+^ mammary epithelial cells are surrounded by the normal stromal microenvironment, this phenotype is suppressed [49, 50].

Several studies of breast cancer have found that chromosomal region 8p22, where Dlc1 maps, is a site of frequent deletions without a loss of heterozygosity, suggesting the location of a haploinsufficient tumor suppressor(s) [1, 51, 52]. Xue et al. have suggested that Dlc1 and other putative tumor suppressor genes on chromosome region 8p22 are haploinsufficient, since they showed heterozygous deletion and reduced expression in tumors, but not total loss of expression [7]. Our results showing that heterozygous loss of Dlc1 can alter mammary morphogenesis and epithelial cell polarity in 3D cultures, suggests that Dlc1 is haploinsufficient for these processes. Previously, we showed that serum free mouse embryo cells homozygous for Dlc1^gt/gt^ showed a significant increase in RhoA activity compared with wild type cells, suggesting that increased RhoA activity is responsible for the loss of polarity [32]. This also suggests that Dlc1 haploinsufficiency may contribute to tumor progression by disrupting epithelial polarity along with oncogene activation. Future studies to address this hypothesis are now in progress.

## Conclusions

This study shows that decreased Dlc1 expression can disrupt mammary epithelial cell polarization, which results in increased mammary ductal branching. This suggests that normally Dlc1 plays a role in maintaining epithelial cell polarity and that it is haploinsufficient.

## Abbreviations

3D: Three dimensional
BSA: Bovine serum albumin
BPE: Bovine pituitary extract
CCAC: Canadian Council on Animal Care
Dlc1: Deleted in Liver Cancer 1
Dlc1^gt/+^: Heterozygous Dlc1^gt/+^ gene-trapped
DAPI: 4′,6-diamidino-2-phenylindole
EGF: Epidermal growth factor
EDTA: Ethylenediaminetetraacetic acid
FITC: Fluorescein isothiocyanate
FAK: Focal adhesion kinase
FBS: Fetal bovine serum
GAPDH: Glyceraldehyde 3-phosphate dehydrogenase
H&E: Hematoxylin-eosin
MEGM: Mammary epithelial growth media
MOI: Multiplicity of infection
PAGE: Polyacrylamide gel electrophoresis
PE: Phycoerythrin
PVDF: Polyvinylidene difluoride
PBS: Phosphate buffered saline
PLC-δ1: Phospholipase C-delta 1
RNA: Ribonucleic acid
RhoGAP: Rho GTPase activating protein
rpm: Revolutions per minute
RhoGEF: RhoA guanine nucleotide exchange factor
RT-qPCR: Reverse transcription quantitative polymerase chain reaction
ROCK2: Rho associated protein kinase 2
SAM2: Sterile α motif
START: StAR related lipid transfer
shRNA: Short hairpin RNA
SDS: Sodium dodecyl sulphate
TEB: Terminal end bud
TBS: Tris buffered saline
WT: Wild type
